# Phase II clinical study of neoadjuvant chemotherapy with CDDP/CPT-11 regimen in combination with radical hysterectomy for cervical cancer with a bulky mass

**DOI:** 10.1007/s10147-016-1008-7

**Published:** 2016-06-24

**Authors:** Tadahiro Shoji, Eriko Takatori, Yoko Furutake, Anna Takada, Takayuki Nagasawa, Hideo Omi, Masahiro Kagabu, Tatsuya Honda, Fumiharu Miura, Satoshi Takeuchi, Seisuke Kumagai, Akira Yoshizaki, Akira Sato, Toru Sugiyama

**Affiliations:** 1 Department of Obstetrics and Gynecology, Iwate Medical University School of Medicine, 19-1 Uchimaru, Morioka, 020-8505 Japan; 2 Department of Obstetrics and Gynecology, National Hospital Organization Kokura Medical Center, Kokura, Japan; 3 Department of Gynecology, Miyama Hospital, Oshu, Japan; 4 Department of Gynecology, Medical Coat Hachinohe West Hospital, Hachinohe, Japan

**Keywords:** Cervical cancer, Bulky mass, NAC, CDDP, CPT-11

## Abstract

**Background:**

We examined the efficacy and safety of neoadjuvant chemotherapy (NAC) with the CPT-11 + CDDP regimen in combination with radical hysterectomy.

**Subjects and methods:**

The subjects were 42 patients with stages IB2 to IIIB squamous cell carcinoma of the uterine cervix with a bulky mass. CDDP at 70 mg/m^2^ was intravenously administered on day 1 and CPT-11 at 70 mg/m^2^ was intravenously administered on days 1 and 8 of a 21-day cycle. In principle, two cycles were administered followed by radical hysterectomy. We examined antitumor efficacy, adverse events, completion rate of radical hysterectomy, operative time, surgical blood loss, progression-free survival (PFS), and overall survival (OS).

**Results:**

The antitumor effect was complete response in 7 patients, partial response in 28, stable disease in 6, and progressive disease in 1; the response rate was 83.3 % (95 % confidence interval, 68.6–93.0). Grade 3 or more severe neutropenia, anemia, and platelet count decreases were noted in 23 (54.8 %), 4 (9.5 %), and 1 (2.4 %) patient, respectively. Grade 3 nausea occurred in 3 patients (7.1 %), vomiting in 1 (2.4 %), and grade 3 febrile neutropenia in 2 (7.1 %). The completion rate of radical hysterectomy was 88.1 %. The median operative time and surgical blood loss were 260 min (range, 210–334) and 500 ml (range, 393–898), respectively. The 5-year PFS rate was 67.2 %, and the 5-year OS rate was 68.0 %. In multivariate analysis, lymph node metastasis before NAC [hazard ratio (HR), 34.88] and non-response to NAC (HR 30.58) were significant prognostic factors.

**Conclusion:**

NAC with the CDDP/CPT-11 regimen achieves a high antitumor efficacy with moderate adverse reactions, allowing safe radical hysterectomy, and is thus considered to be a useful therapeutic method that can improve prognosis.

## Introduction

Therapeutic methods for stage IB2 to IIB cervical cancer with a bulky mass differ between Japan and Western countries. Based on the results of large-scale randomized studies and meta-analyses, concurrent chemoradiotherapy (CCRT) is recommended as the standard treatment in Western countries [1–5]. However, an approach using neoadjuvant chemotherapy (NAC) is widely applied clinically in Japan, South Korea, China, and Italy [6]. Irinotecan (CPT-11), a drug developed in Japan, is reportedly useful as monotherapy or in combination with cisplatin (CDDP) for recurrent cervical cancer [7, 8]. We previously reported the efficacy and safety of a treatment regimen involving a 28-day cycle of CDDP administered on day 1 and CPT-11 on days 1, 8, and 15 [9].

From the aspect of reducing the time to surgical therapy as the primary treatment, we conducted a phase II clinical study of NAC in combination with radical hysterectomy with a dosing schedule employing a 21-day cycle with a higher than usual CDDP dose intensity.

## Subjects and methods

### Subjects

The subjects were 42 patients with stages IB2 to IIIB squamous cell carcinoma of the uterine cervix with a bulky mass. These patients gave informed consent and were scheduled to undergo radical hysterectomy during the period from June 2002 to March 2014.

### Justification for the target sample size

We previously reported that the response rate to the CPT-11/CDDP regimen [CPT-11 60 mg/m^2^ (days 1, 8, 15), CDDP 70 mg/m^2^ (day 1) q28 days] was 59 % [9]. Accordingly, the threshold response rate was set at 50 %, because it would not be worthwhile to use this strategy in a clinical setting if the response rate was significantly lower than 50 %. The response rates to platinum-based NAC in patients with cervical cancer reportedly range from 76 % to 95 % [6, 10, 11]. Accordingly, the expected response rate was set at 80 %, anticipating that the response rate to this regimen would be 80 %. The number of patients required was calculated to be 36 based on a presumed binominal distribution with a threshold value of 59 %, expected response rate of 80 %, and two-sided α-level of 0.05 and β-level of 0.2 (1 − β = 0.8). The number of planned subjects was thus set at 40 patients, taking into account the possibility of a few patients becoming ineligible or dropping out.

### Inclusion criteria

The following set of inclusion criteria was employed for selection of study subjects. (1) Histologically verified squamous cell carcinoma of the uterine cervix; (2) age more than 20 years and less than 70 years; (3) locally advanced stage IB2 to IIIB; (4) Eastern Cooperative Oncology Group (ECOG) performance status (PS) 0–2; (5) initially treated case; (6) presence of a magnetic resonance imaging (MRI)-measurable bulky mass in the uterine cervix; (7) hematological and blood biochemical findings meeting the following criteria (WBC count ≥4,000/mm^3^; neutrophil count ≥2,000/mm^3^; platelet count ≥100,000/mm^3^; hemoglobin ≥10.0 g/dl; AST and ALT levels ≤2 times the upper limit of normal reference range at study site; serum total bilirubin level ≤1.5 mg/dl; serum creatinine ≤1.5 mg/dl; and creatinine clearance ≥60 ml/min); (8) life expectancy ≥6 months; and (9) written informed consent personally given by the subject.

### Exclusion criteria

Exclusion criteria were prescribed as follows. (1) Patients with overt infection; (2) patients with a serious complication (e.g., cardiac disease, poorly controlled diabetes mellitus, malignant hypertension, bleeding tendency); (3) patients with active multiple cancer; (4) patients with interstitial pneumonia or pulmonary fibrosis; (5) patients with effusions; (6) patients with a history of unstable angina or myocardial infarction within 6 months after registration, or with a concurrent serious arrhythmia requiring treatment; (7) patients for whom treatment with cisplatin and irinotecan is contraindicated; (8) patients with (watery) diarrhea; (9) patients with intestinal paralysis or ileus; (10) pregnant women, nursing mothers, or women wishing to become pregnant; (11) patients with a history of serious drug hypersensitivity or drug allergy; and (12) patients who were inadequate for safe conduct of this study as judged by the attending physician.

### Neoadjuvant chemotherapy

CDDP at 70 mg/m^2^ was intravenously administered on day 1 and CPT-11 at 70 mg/m^2^ was intravenously administered on days 1 and 8 of a 21-day cycle. In principle, two cycles were administered followed by radical hysterectomy.

### Dose modification criteria

1. Criteria for CPT-11 dose skip. The CPT-11 dose on day 8 will be skipped if hematological test values within 2 days before day 8 fail to fulfill the following criteria: (1) neutrophil count ≥1,000/mm^3^, and (2) platelet count ≥75,000/mm^3^.
2. Criteria for initiation of the second cycle. Initiation of the second cycle will be postponed up to a maximum of 2 weeks if hematological test values within 2 days before the scheduled second cycle initiation day fail to fulfill the following criteria: (1) neutrophil count ≥1,500/mm^3^, (2) platelet count ≥75,000/mm^3^, and (3) serum creatinine ≤1.5 mg/dl.

### Dose reduction criteria

The doses of cisplatin and irinotecan will be reduced to 70 and 60 mg/m^2^, respectively, in the second course for patients for whom any of the following signs of toxicity is noted in the first cycle: (1) grade 4 neutropenia persisting for ≥7 days, (2) febrile neutropenia persisting for ≥4 days, (3) grade 4 thrombocytopenia, (4) grade 3 thrombocytopenia with hemorrhage, and (5) grade ≥3 nonhematological toxicity excluding nausea, vomiting, appetite loss, fatigue, and hair loss.

### Supportive therapy

Therapeutic administration of granulocyte-colony-stimulating factor (G-CSF) preparations was undertaken when grade 4 neutropenia was noted in the first cycle. In the second cycle and thereafter, prophylactic use of the preparation in patients with grade 3 neutropenia was acceptable if grade 4 neutropenia had been noted in the first cycle. Antiemetics were used for preventive purposes.

### Endpoints/variables

The primary endpoint was antitumor efficacy, and the secondary endpoints were adverse events, completion rate of radical hysterectomy, operative time, surgical blood loss, progression-free survival (PFS), and overall survival (OS). Antitumor efficacy, PFS, and OS were also calculated by stage.

For the determination of antitumor efficacy, MRI was performed after the completion of course 1 and course 2, using MRI images before treatment as the baseline. Antitumor efficacy was determined using the Response Evaluation Criteria in Solid Tumors (RECIST) version 1.1, with response being the best evaluation. Adverse events were evaluated according to the National Cancer Institute Common Toxicity Criteria (NCI-CTCAE) version 4.0.

### Main treatment

Stage Ib2–IIIb patients were subjected to a radical hysterectomy unless the antitumor response was progression of disease or up-stage progression. A radical hysterectomy was performed on stage IIIb patients with a down-stage progression. As a rule, lymph node dissection included the lymph nodes within the pelvis (external iliac nodes, internal iliac nodes, common iliac nodes, suprainguinal nodes, parametrial nodes, and obturator nodes). Concurrent chemoradiation therapy (CCRT) was carried out for patients whose conditions were inoperable.

### Postoperative adjuvant therapy

Patients with a positive surgical margin, metastatic lymph nodes, infiltration to the parametrium, and/or vascular invasion, as demonstrated by pathological examination of the resected specimens, underwent postoperative irradiation, chemotherapy or CCRT. Before 2008, irradiation was performed as postoperative adjuvant therapy, whereas chemotherapy was performed from 2008 onward. However, CCRT was performed for patients who had multiple lymph node metastases and/or infiltration to the parametrium.

### Statistical analysis

Progression-free survival and overall survival were calculated from the date of start of NAC, to the documented date of progression, death, or last follow-up, whichever occurred first. Impact of surgery result on survival was assessed by constructing Kaplan–Meier curves with a log-rank test. Cox regression analyses were performed to assess the prognostic factors on survival. All reported significance was two tailed at a level of 0.05.

## Results

### Clinical characteristics

Among 43 patients enrolled, 1 was diagnosed as having glassy cell carcinoma based on the postoperative histopathological examination. Therefore, we analyzed the other 42 patients. The median age was 45 years (range, 25–63). The performance status was 0 in 37 patients and 1 in 5, and clinical progression was stage IB2 in 9 patients, stage IIA2 in 2, stage IIB2 in 27, and stage IIIB in 4. Histological subtypes were keratinizing type in 11 patients and non-keratinizing type in 31. Computed tomography before NAC showed lymph node metastasis in 16 patients and no lymph node metastasis in 26. The tumor size was less than 5 cm in 18 patients and 5 cm or more in 24 by MRI before NAC. Thirteen patients were positive and 29 patients were negative for pathological lymph node metastasis. Postoperative treatments were radiation therapy in 13 patients, chemotherapy in 15, and chemoradiotherapy in 3; no postoperative treatment was given to 9 patients (Table 1).

**Table 1:** Patient characteristics

Characteristic	*n* = 42
Age (years)	
Median (range)	45 (25–63)
PS	
0/1	37/5
FIGO stage	
1/11/III	9/29/4
Histological type	
Keratinizing type/non-keratinizing type	11/31
Preoperative lymph node metastasis	
Positive/negative	16/26
Tumor diameter	
>5 cm/<5 cm	24/18
Pathological lymph node metastasis	
Positive/negative	13/29
Postoperative treatment	
None	9
Radiation	13
Chemotherapy	15
Chemoradiotherapy	3

### Response

The antitumor effect was complete response (CR) in 7 patients, partial response (PR) in 28, stable disease (SD) in 6, and progressive disease (PD) in 1, and the response rate was 83.3 % [95 % confidence interval (CI), 68.6–93.0]. Response rates by progression were 100 % for stage I, 82.8 % for stage II, and 50.0 % for stage III (Table 2).

**Table 2:** Response (*n* = 42)

	CR	PR	SD	PD	Objective response (CR + PR)
Total (*n* = 42)	7	28	6	1	35 (83.3 %)
Stage l (*n* = 9)	2	7	0	0	9 (100 %)
Stage ll (*n* = 29)	2	20	4	1	24 (82.8 %)
Stage lll (*n* = 4)	1	1	2	0	2 (50.0 %)

*CR* complete response, *PR* partial response, *SD* stable disease, *PD* progressive disease

### Adverse events

Grades 3 and 4 neutropenia were observed in 11 (26.2 %) and 12 (28.6 %) patients, respectively. Grade 3 or more severe anemia was noted in 4 patients (9.5 %); among them, a patient with grade 4 anemia required a blood transfusion. Grade 3 or more severe decreases in platelet counts were observed in 1 patient (2.4 %). As grade 3 or more severe nonhematological toxicity, grade 3 nausea occurred in 3 patients (7.1 %), vomiting in 1 (2.4 %), and grade 3 febrile neutropenia in 2 (7.1 %) (Table 3).

**Table 3:** Adverse events (*n* = 42)

	Grade 1	Grade 2	Grade 3	Grade 4	Grade >3
Hematological toxicity					
Leukopenia	8	20	8	3	11 (26.2)
Neutropenia	4	14	11	12	23 (54.8)
Anemia	17	20	3	1	4 (9.5)
Thrombocytopenia	10	2	1	0	1 (2.4)
Nonhematological toxicity					
Nausea	25	9	3	0	3 (7.1)
Vomiting	21	7	1	0	1 (2.4)
Diarrhea	4	2	0	0	0 (0)
Creatinine	2	0	0	0	0 (0)
Febrile neutropenia	0	0	2	0	2 (4.8)

Among the total 42 patients, 40 (95.2 %) completed chemotherapy as scheduled. The administration of CPT-11 on day 8 of cycle 2 was skipped in the remaining 2 patients. Grade 3 nausea persisted in these 2 patients; although it did not satisfy the skip criteria, in both cases the primary physician decided to skip the administration. The administration in cycle 2 was postponed in 3 patients (10.7 %) because neutrophil counts did not satisfy the therapy initiation criteria; however, all these 3 patients were able to receive treatment within 7 days. In 2 patients (10.0 %) in whom febrile neutropenia continued for 4 days or more, the doses of CPT-11 and CDDP were reduced from 70 to 60 mg/m^2^.

### Operative details

**Fig. 1:**
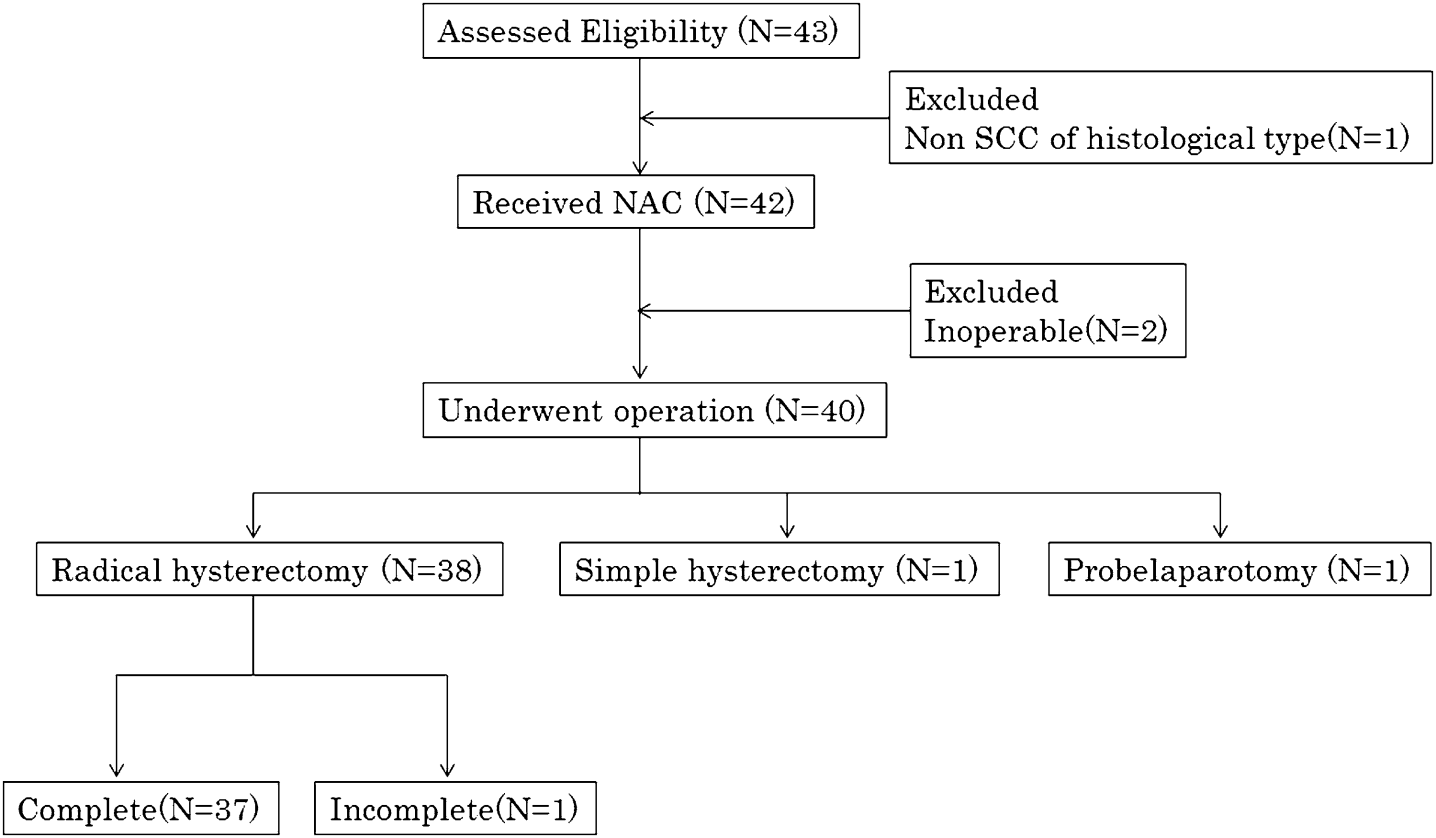
Operation consort diagram. *SCC* squamous cell carcinoma, *NAC* neoadjuvant chemotherapy

Among the total 42 patients, 2 with stage IIIB had inoperable disease. Forty patients underwent surgery. Radical hysterectomy was performed for 38 patients, but in 1 of them surgery was incomplete because of difficulty in resecting the lymph node metastasis. Simple total hysterectomy and exploratory laparotomy were performed in 1 patient each. The operable rate of surgery was 95.2 %; completion rate of radical hysterectomy was 88.1 % (Fig. 1). Median number of lymph nodes dissected was 22 (range, 3–49); median operative time was 260 min (range, 210–334); median blood loss was 500 ml (range, 393–898). Blood transfusions were given to 6 of 40 patients (15.0 %). The median time from surgery to discharge was 21 days (range, 16–26) (Table 4).

**Table 4:** Operative details

Characteristic	*n* = 42
Type of surgery	40
RH	38
TAH	1
Probe laparotomy	1
Inoperable	2
Operation time (min)	
Median (IQR)	260 (210–334)
Blood loss (ml)	
Median (IQR)	500 (393–898)
Blood transfusion	
Yes	6
No	34
Time from surgery to discharge (Days)	
Median (IQR)	21 (16–26)
Pathological lymph node metastasis	
Positive	13
Negative	29

*RH* radical hysterectomy, *TAH* total abdominal hysterectomy, *IQR* interquartile range

### PFS and OS analysis

Of the 38 patients who underwent radical hysterectomy (excluding 1 patient who underwent probelaparotomy), 10 patients (26.3 %) developed tumor recurrence, that is, recurrence was noted in 1 (50.0 %) of the 2 patients who received postoperative CCRT, 2 (13.3 %) of the 15 patients who received postoperative chemotherapy, 5 (41.7 %) of the 12 patients who received postoperative radiotherapy, and 2 (22.2 %) of the 9 patients who did not receive postoperative adjuvant therapy (Table 5). Tumor recurrence in the lymph nodes was noted in 4 patients (affecting the paraaortic lymph nodes in all 4 cases and accompanied by metastasis to the subclavicular lymph nodes in 1 case). Of the 4 patients unable to undergo radical hysterectomy, 1 patient underwent CCRT after a simple total hysterectomy. CCRT was used for the patient who underwent probelaparotomy, and CCRT and radiation were used for the 2 patients judged unable to receive post-NAC surgery, but all 3 of these patients developed intrapelvic recurrence.

**Table 5:** Details of recurrent patients after radical hysterectomy (*n* = 38)

Adjuvant therapy	Number	Rate (%)	Recurrent sites
Chemoradiotherapy (*n* = 2)	1	50.0	PAN (l)
Chemotherapy (*n* = 15)	2	13.3	PAN (l), pelvic cavity (l)
Radiation (*n* = 12)	5	41.7	PAN (2), subclavicular lymph node (l), pelvic cavity (3)
No therapy (*n* = 9)	2	22.2	Vaginal stump (2)

*PAN* paraaortic lymph node

**Fig. 2:**
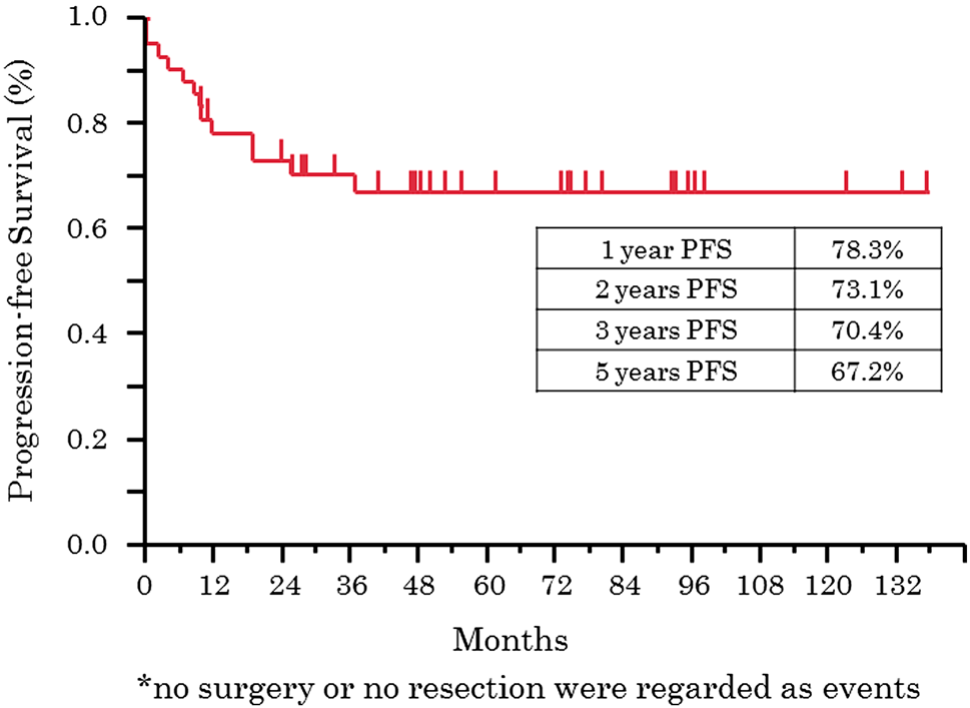
Kaplan–Meier plot of progression-free survival (*n* = 42)

**Fig. 3:**
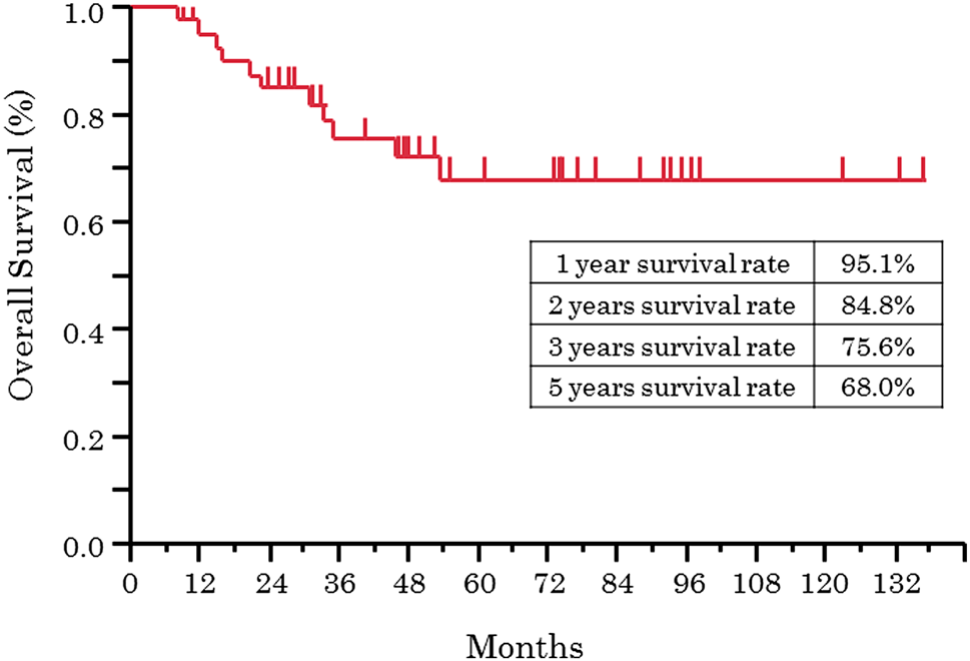
Kaplan–Meier plot of overall survival (*n* = 42)

The median follow-up period for 42 patients was 45 months (range, 8–143), and the 5-year PFS rate and 5-year OS rate were 67.2 % and 68.0 %, respectively. As to progression, the 5-year PFS rates for patients at stages I, II, and III were 53.3 %, 78.4 %, and 25.0 %, respectively, and the 5-year OS rates were 53.3 %, 79.2 %, and 25.0 %, respectively (Figs. 2, 3). In multivariate analysis of OS, lymph node metastasis before NAC [hazard radio (HR), 34.88; *p* = 0.0031] and response of NAC (HR, 30.58; *p* = 0.0014) were extracted as significant prognosis factors (Table 6).

**Table 6:** Multivariate analysis of treatment-related factors for overall survival (OS)

Factor		Hazard ratio	95 % CI	*p* value
PS	0/1	0.11	0.002–2.693	0.1858
FIGO stage	Stage I, II/III	5.75	0.224–90.468	0.2435
Tumor diameter	>5/<5	0.25	0.009–3.177	0.2788
Pre-NAC lymph node metastasis	Positive/negative	34.88	2.969–1152.9	0.0031
Tumor response	CR, PR/SD, PD	30.59	3.675–447.9	0.0014
Pathological lymph node metastasis	Positive/negative	1.21	0.182–7.932	0.836
Postoperative treatment	Yes/no	6.81	0.718–174.323	0.0983

*NAC* neoadjuvant chemotherapy

## Discussion

Clinical studies of NAC for various forms of cancer are underway, but as yet there is little evidence of the usefulness of NAC in the field of gynecology including cervical, endometrial, and ovarian cancers. The clinical significance of NAC for cervical cancer is that the following can be expected: (1) tumor size reduction, which improves operative curability and safety and thus expands the surgical indications; and (2) inhibition of distant metastasis based on systemic effects being exerted on latent and lymph node micrometastatic lesions. As such, NAC for cervical cancer in Japan is currently performed to improve operative curability and safety as well as to increase the indications for surgery. However, there are no reports indicating that NAC prolongs survival.

A key drug for progressive recurrent cervical cancer is CDDP; the results of a randomized study by the Gynecologic Oncology Group (GOG) demonstrated the usefulness of paclitaxel and topotecan in addition to CDDP, and dual therapy with CDDP has been recommended for recurrence and progressive cancer [12, 13]. Topotecan is widely used in Western countries, whereas mainly CPT-11, with a mechanism of action based on the same DNA type I topoisomerase inhibitory action, is used in Japan.

In a study of combination therapy with CDDP [CDDP 60 mg/m^2^ (day 1) + CPT-11 60 mg/m^2^ (days 1, 8, 15)], the response rate in patients with recurrent/progressive cervical cancer was 59.9 %, and the response rate when this regimen was administered as NAC was 78 % [6, 9]. To reduce the time to surgical therapy, the primary treatment, we conducted a phase II clinical study of NAC in combination with radical hysterectomy with a dosing schedule involving a 21-day cycle with a higher than usual CDDP dose intensity. The response rate in 42 patients was 83.3 %, better than the therapeutic results of Sugiyama et al. Notably, a response rate of 86.8 % was obtained in 38 patients with stage IB2 to IIB disease. However, the response rates were 50.0 % in 4 stage IIIB patients, 50 % in operated patients, and 25 % in patients who had completed radical hysterectomy. Our results indicate that NAC plus radical hysterectomy is not useful in stage III patients.

With regard to adverse events, the incidence of grade 3 or more severe neutropenia was 54.8 %, although this adverse event could be managed with a G-CSF agent. In all cases with diarrhea characteristic of CPT-11, the severity was grade 2 or lower. A single dose of CPT-11 had to be reduced in this regimen because of administration in divided doses on days 1 and 8, thereby preventing grade 3 or more severe diarrhea.

In 40 patients undergoing NAC plus radical hysterectomy, median operative time was 260 min and median surgical blood loss was 500 ml, similar to those findings in studies of radical hysterectomy for patients with stage IB2 to IIB with a bulky mass reported by He et al. and Wang et al. [14, 15]. No serious postoperative complications, such as intestinal obstruction and thrombosis, occurred. Accordingly, this study demonstrated the safety of NAC plus radical hysterectomy, with no major adverse effects for patients.

We compared the treatment outcomes in our 38 IB2–IIB cases with the results reported by Uegaki et al. [16]. The response rate for our cases was 86.6 %, comparable to the rate reported by Uegaki et al. (86.2 %). The 5-year PFS (73.3 % vs. 62.2 %) and 5-year OS (78.9 % vs. 74.9 %) were better in our cases than in the cases reported by Uegaki et al. Comparisons were also made with respect to adjuvant therapy and recurrence sites. According to the report by Uegaki et al., recurrence was noted in 21 (32.3 %) of the 65 cases, and 15 of these 21 cases had received adjuvant therapy, but recurrence in the pelvic cavity was observed in only 4 cases (the other cases of recurrence had remote metastasis, including metastasis to the paraaortic lymph nodes). Of the 38 patients we managed, 10 patients (26.3 %) developed recurrence, including 8 patients who received adjuvant therapy (4 patients with intrapelvic recurrence and the other 4 patients with metastasis to lymph nodes, including the paraaortic lymph nodes). Postoperative adjuvant therapy by means of CCRT or radiation was performed in 27 (93.1 %) of the 29 cases reported by Uegaki et al. and 14 (50.0 %) of the 28 cases managed by us. Postoperative CCRT or radiation allowed control of intrapelvic recurrence to some extent, whereas postoperative chemotherapy tended to reduce the risk of remote recurrence. These results suggest that the site of recurrence depends on the type of postoperative adjuvant therapy. Furthermore, as recurrence was also observed in cases that did not receive postoperative adjuvant therapy, it seems advisable to perform postoperative adjuvant therapy in all cases of bulky tumors.

Six randomized clinical trials (1036 patients) including Study JCOG102 in Japan were analyzed in Cochrane Reviews in 2010, and NAC in combination with surgical therapy reportedly improved the disease-free survival rate as compared with initial surgery (HR, 0.76; *p* = 0.01). NAC plus radical hysterectomy was thus suggested to improve patient prognosis [17]. According to a multivariate analysis of OS, clinical stages, lymph node metastasis before NAC, and antitumor effects are significant prognostic factors. Therefore, it was suggested that NAC plus radical hysterectomy, for patients other than those with lymph node metastasis before NAC, may further improve the therapeutic effects of NAC. That is, the prognosis of patients with stage IB2 to IIB cervical cancer with a bulky mass might be improved by hysterectomy alone in those who have lymph node metastasis before NAC, and by NAC plus radical hysterectomy in those without lymph node metastasis. The next step we should take would be implementation of a phase II clinical study on the use of NAC plus radical hysterectomy plus adjuvant therapy, with details of postoperative adjuvant therapy set forth in advance, in patients having a bulky tumor (clinical stage IB2–IIB). The results from such a study would provide important data for planning a phase III clinical study on a national scale of NAC plus radical hysterectomy plus adjuvant therapy versus radical hysterectomy plus adjuvant therapy versus CCRT.

## Conclusions

The results of this study suggest that NAC, that is, the CDDP/CPT-11 regimen, in combination with radical hysterectomy exerts high antitumor efficacy with manageable adverse reactions. Thus, NAC may facilitate safe radical hysterectomy. This treatment strategy is considered to be therapeutically useful, and an improved prognosis can also be expected. Therefore, we add our evidence of the usefulness of NAC plus radical hysterectomy in Japan to the world literature on cervical cancer treatment.
